# Integrating Gene Correction in the Reprogramming and Transdifferentiation Processes: A One-Step Strategy to Overcome Stem Cell-Based Gene Therapy Limitations

**DOI:** 10.1155/2016/2725670

**Published:** 2016-12-15

**Authors:** Seo-Young Lee, Sun-Ku Chung

**Affiliations:** Medical Research Division, Korea Institute of Oriental Medicine, 1672 Yuseong-daero, Yuseong-gu, Daejeon 34054, Republic of Korea

## Abstract

The recent advent of induced pluripotent stem cells (iPSCs) and gene therapy tools has raised the possibility of autologous cell therapy for rare genetic diseases. However, cellular reprogramming is inefficient in certain diseases such as ataxia telangiectasia, Fanconi anemia, LIG4 syndrome, and fibrodysplasia ossificans progressiva syndrome, owing to interference of the disease-related genes. To overcome these therapeutic limitations, it is necessary to fundamentally correct the abnormal gene during or prior to the reprogramming process. In addition, as genetic etiology of Parkinson's disease, it has been well known that induced neural stem cells (iNSCs) were progressively depleted by LRRK2 gene mutation, LRRK2 (G2019S). Thus, to maintain the induced NSCs directly derived from PD patient cells harboring LRRK2 (G2019S), it would be ideal to simultaneously treat the LRRK2 (G2019S) fibroblast during the process of TD. Therefore, simultaneous reprogramming (or TD) and gene therapy would provide the solution for therapeutic limitation caused by vulnerability of reprogramming or TD, in addition to being suitable for general application to the generation of autologous cell-therapy products for patients with genetic defects, thereby obviating the need for the arduous processes currently required.

## 1. Introduction

Since their discovery in 2006, induced pluripotent stem cells (iPSCs) have been considered to be highly useful resources for cell-replacement therapy as well as for studying human disease. Thus, iPSCs are expected to be applicable to the treatment of a broad range of diseases, including neurological disorders, hematological abnormalities, spinal cord injury, heart disease, diabetes, and arthritis [1, 2]. Several groups have already reported the generation of a variety of iPSCs derived from patients with genetic disorders such as amyotrophic lateral sclerosis, familial dysautonomia, spinal muscular atrophy, adenosine deaminase deficiency-related severe combined immunodeficiency, dyskeratosis congenita, Shwachman-Bodian-Diamond syndrome, leopard syndrome, Gaucher disease type III, Duchenne muscular dystrophy, Becker muscular dystrophy, Timothy syndrome, Parkinson's disease (PD), Huntington's disease, Hutchinson-Gilford progeria syndrome, juvenile-onset type 1 diabetes mellitus, Down syndrome, Rett's syndrome, and Lesch-Nyhan syndrome [3–11]. Fortunately, these disease-related iPSCs were generated without the negative influences of genetic mutations. Although disease-related genes may potentially exert adverse effects on the reprogramming process, leading to poor reprogramming efficiency and inhibitory maintenance, this is not considered a crucial concern unless the gene mutations are so severe to bring about very early embryonic lethality. Nevertheless, even for inherited genetic disorders without severe lethality in the embryonic development stage, certain disease-related genes can seriously impede the reprogramming process or impair the maintenance of iPSCs, which has been observed in cases of ataxia telangiectasia (A-T) [12], Fanconi anemia (FA) [13, 14], LIG4 syndrome [15], and fibrodysplasia ossificans progressiva (FOP) syndrome [16, 17]. Therefore, it is important to generate gene-corrected iPSCs to avoid the potential of reprogramming impairment by interference of a defective gene. To achieve this, it is necessary to genetically treat the iPSCs from the somatic cell phase prior to reaching the impaired iPSCs stage.

Another strategy for cell-replacement therapy is transdifferentiation (TD), also known as direct reprogramming, which is a process in which lineage-specific cell types are directly derived from somatic cell types, thereby bypassing the pluripotency stage. TD possesses several advantages such as the rapid generation of specific cell types as well as avoidance of the teratoma formation caused by the intrinsic characteristics of iPSCs. However, the TD-mediated lineage-specific cells may also be impaired by disease-related genes. A prime example of this effect is the G2019S mutant of leucine-rich repeat kinase 2 (LRRK2), which leads to nuclear disruption in induced neural stem cells (iNSCs) and has been detected in brain slices of PD patients [18]. Although NSCs can be successfully generated from iPSCs with the LRRK G2019S mutation, they are completely depleted after several passages due to abnormal interactions between LRRK2 (G2019S) and lamin B1 protein, which is anchored to the inner nuclear membrane and is involved in breaking the framework of the nuclear envelope [18]. Thus, this interaction would have a negative effect on the formation of NSCs directly derived from PD patient cells via the TD process. Therefore, along with the concurrent reprogramming and gene-correction approach, the* LRRK2* (G2019S) gene would be fundamentally treated using gene-correction tools during the TD process rather than at the NSC stage in which the cells will still harbor the mutant gene.

In this review, we highlight the challenges facing current iPSC-based therapy approaches and introduce the one-step gene-correction and reprogramming approach as the solution to overcome iPSC-based gene therapy limitations. And we also propose adoption of an ideal gene therapy approach that combines the gene-correction process with the TD process, focusing on the example of the pathogenic LRRK2 mutant (G2019S), which progressively depletes neural stem cells in Parkinson's disease.

## 2. Genetic Defects That Affect Reprogramming

### 2.1. DNA Repair Defects Affecting the Reprogramming Process

One of the main challenges of the reprogramming process is interference owing to genome instability or apoptosis induction [20, 21]. During this process, p53 protein, a crucial monitor of genome integrity, accumulates in response to the ectopic overexpression of reprogramming factors [22]; thus, p53 strictly regulates the reprogramming of somatic cells and can impede this process overall. Accordingly, temporary reduction in the activity of p53 can successfully enhance the reprogramming efficiency by no less than 100 times the original value [23–26], owing to the lack of concern about apoptosis and DNA damage [27]. As one of the kinases that activates p53 in the DNA damage response, the ataxia-telangiectasia mutated (*ATM*) gene also plays a pivotal role in regulating genome stability. Chromosomal instability caused by* ATM *mutation results in the development of A-T, a rare inherited disorder that is characterized by motor neurodegeneration, leukemia, and premature ageing [28]. The broad phenotypes associated with A-T have an additional influence on reprogramming efficiency. Consequently, the efficiency of iPSCs generated from A-T fibroblasts is extremely low, at about only 4%, in A-T homozygote cells compared with normal cells [12, 29].

FA syndrome is another rare inherited disease characterized by chromosomal instability syndromes such as aplastic anemia, leukemia, and breast or ovarian cancers [30]. Thus, genes associated with FA are also involved in DNA repair, especially DNA interstrand crosslink repair, and therefore mutations of these genes have the potential to impair the reprogramming process. To develop an FA-iPSC model, some groups have attempted to generate iPSCs from patients with FA who harbor* FANCA* or* FANCD2*, or from an FA mouse model with* FANCA*,* FANCC*, or* FANCD1/BRCA2* (breast and ovarian cancer susceptibility protein 2) [13, 31, 32]. Although the FA-related genes do not induce early developmental lethality, iPSCs cannot be successfully generated until these genes are complementary to patient-derived fibroblasts [14]. Moreover, when mouse iPSCs were generated from FA mouse fibroblasts, the reprogramming efficiency was also found to be reduced or impaired [13, 20, 32]. BRCA1 coopts several FA proteins, including BRCA2 [33, 34]. The complex formed by these proteins results in BRCA1 manifesting a similar phenotype to BRCA2, indicating that reprogramming from BRCA1-deficient mouse embryonic fibroblasts (MEFs) is also impaired in a similar manner to that observed in BRCA2-deficient MEFs [20]. Meanwhile, as one of the interaction proteins of BRCAs, RAD51 plays a key role in the DNA repair process through homologous recombination. Similar to the low reprogramming efficiency induced by the functional loss of* BRCA* genes, the silencing of* RAD51* gene expression also seriously reduced reprogramming efficiency [20].

Besides a DNA repair system involving ATM-, FA-, BRCA-, or RAD51-dependent homologous recombination (HR), nonhomologous end joining (NHEJ) is also an important DNA repair system involving direct ligation of the end of the DNA strand break region. In this pathway, DNA ligase IV (encoded by the* LIG4* gene) participates in repairing double-stranded breaks during the final step of NHEJ and V(D)J recombination [35, 36]. Mutations in the* LIG4* gene are associated with LIG4 syndrome, which is characterized by leukemia, immunodeficiency, and developmental retardation. In addition, although the consequences of this hypomorphic mutation of the* LIG4* gene are less severe than the mouse embryonic lethality caused by the disrupted* LIG4* gene, the reprogramming efficiency in cells derived from patients carrying the* LIG4* gene mutation is significantly lower than that of normal control cells [15].

The examples highlighted above demonstrate that several chromosomal instability-related syndromes are associated with limitations in reprogramming, implying a strong link between reprogramming efficiency and genome instability (Table 1).

### 2.2. The Constitutively Activated Bone Morphogenetic Protein (BMP) Signaling Pathway Affects Reprogramming

As members of transforming growth factor-beta, BMPs bind to BMP type II receptor, and then BMP-bound type II receptor kinase phosphorylates BMP type I receptor to activate Smad proteins, including Smad 1/5/8, which play major roles in bone formation [37]. In addition to their roles in cellular differentiation, BMPs also regulate the histone H3 lysine 9 methylation that functionally impedes somatic cell reprogramming [38]. Thus, activation of the BMP signaling pathway prevents reprogramming beyond the intermediate pre-iPSCs stage [38]. Indeed, fibroblasts derived from patients with FOP syndrome that harbor an intrinsic abnormal BMP type I receptor also exhibit atypical reprogramming as well as inhibited self-renewal following incomplete reprogramming [16, 17]. FOP is directly caused by mutations of the activin A receptor, type 1 (*ACVR1*) gene, which result in the synthesis of an abnormal activin receptor-like kinase 2 (ALK2) protein, leading to constitutive activity of BMP type I receptor and aberrant heterotopic ossification [37]. ALK2 has been shown to beneficially contribute to the efficacy of iPSCs generation only in the early phase of the reprogramming process, whereas constitutive ALK2 activation hampers iPSCs generation after the early phase [16]. In addition to the low reprogramming efficiency, mutant ALK2-iPSCs (mALK2-iPSCs) phenotypically exhibit weak alkaline phosphatase activity, indicating incomplete reprogramming, as well as a tendency to differentiate into osteoblasts and mineralize, with high expression of representative osteogenic marker genes [17]. Therefore, mALK2-iPSCs cannot be efficiently stabilized or maintained unless the abnormal ALK2 protein is genetically treated or functionally weakened using ALK2 inhibitors.

## 3. Gene-Correction Tools and Limitations of iPSC-Based Gene Therapy

There are still many kinds of diseases that are not confirmed in reprogramming efficiency. And some of them would face the limitation of iPSC-based gene therapy due to the inefficient reprogramming affected by gene defect. However, as highlighted with the examples in the preceding sections, to overcome these limitations, it is necessary to completely remove the causal gene from the patient-derived somatic cells prior to the generation of iPSCs, or during the reprogramming process stage. Viral vector-mediated* FA* gene therapy is the first example to overcome the limitation of iPSC-based gene therapy. Owing to the poor reprogramming ability of cells derived from patients with several FA-related disorders, treatment strategies have been initiated using recombinant viral vector-mediated gene therapy, thereby enabling the functional restoration of nonfunctional FA proteins. It is crucial to deliver the exogenous FA gene prior to reprogramming, as the nonfunctional FA gene impairs the reprogramming of patient-derived fibroblasts [13, 14]. However, there are several hurdles to overcome the possibility of silence of the expression of normal gene delivered by viral vector, as well as simplifying the procedure to avoid the requirement of the numerous cumbersome steps involved, such as sorting out viral-genome-integrated cells, and then subsequently reprogramming based on the sorted cells. And although loss of a functional FA gene can be simply complemented by a normal exogenous FA gene, the viral vector-mediated gene therapy cannot be generally applied to the gain of functional gene, such as* ACVR1* c.617G>A, which constitutively activates the Smads signaling pathway as a dominant effect. That is, in order to overcome the limitation of iPSC-based gene therapy as the dominant effect of this mutation, a fundamental treatment must be applied to replace the mutated base with the wild type, simply not complemented by the normal gene.

**Figure 1:**
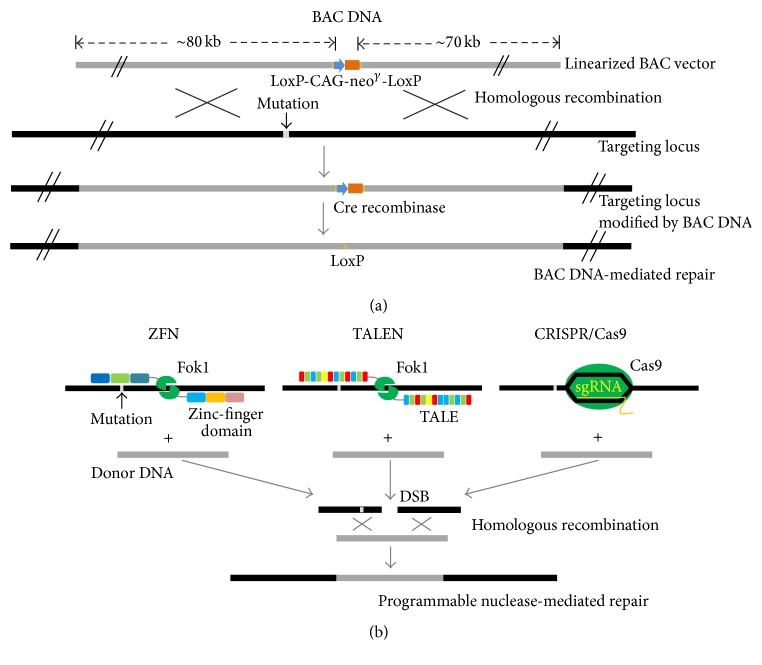
Mode of gene correction by gene-editing tools. (a) The image is drawn on the basis of the previous report [19]. The selection cassette (CAG-Neo^*γ*^) is inserted into the noncoding regions of the normal BAC DNA by recombineering. The lengths of flanking homology arms are indicated. The gene harboring a mutation base is replaced with the normal BAC DNA by homologous recombination. The selection cassette is removed by Cre recombinase. (b) The programmable nuclease modes, such as ZFN, TALEN, or CRISPR/Cas9, induce DNA double-strand break (DSB) at the target locus. The mode of gene correction of programmable nucleases repairs DSB with the donor DNA, which is additionally introduced.

This can best be accomplished by combining the benefits of several precise genome-editing tools such as powerful molecular scissors, including zinc-finger nucleases (ZFNs) [39–41], transcription activator-like effector nucleases (TALENs) [42, 43], RNA-guided endonucleases from the microbial clustered regularly interspaced short palindromic repeat-Cas9 (CRISPR-Cas9) nuclease systems [44–46], or bacterial artificial chromosome- (BAC-) based HR [19], enabling the target genes to be effectively inserted, deleted, or replaced in the genome as needed [17]. To date, the advantages and disadvantages of the ZFN, TALEN, and CRISPR/Cas9 systems have been comprehensively assessed [47]. However, the BAC-based HR system is not popular, despite showing high targeting efficiency for gene correction. The mode of BAC-based HR is by direct recombination of its own huge homologous arm, approximately 70–80 kb in length, at the targeting locus [19, 48, 49]; thus, this method does not require additional donor DNA (Figure 1(a)). By contrast, because the programmable nuclease modes, including ZFN, TALEN, and CRISPR/Cas9, merely induce DNA double-stranded breaks at the target sequence, donor templates are additionally required to effectively mediate gene correction with homology-directed repair (Figure 1(b)).

To effectively apply gene-editing tools to iPSCs, the cells must be resistant to the stressful conditions required in the processes, including trypsinization or transfection. In general, the ROCK inhibitor Y-27632 has been shown to enhance the survivability of single cells dissociated by trypsin, helping to maintain the self-renewal capacity of the cells [50]. Nevertheless, Y-27632 treatment could not prevent mALK2-iPSCs from spontaneously differentiating under trypsinization and transfection, despite enhancing the viability of the cells [17]. Thus, we will suggest the challenges facing iPSC-based therapy approach in the next section.

## 4. Strategy to Overcome the Limitations of Stem Cell-Based Gene Therapy 

### 4.1. Combined Reprogramming and Gene Correction

**Figure 2:**
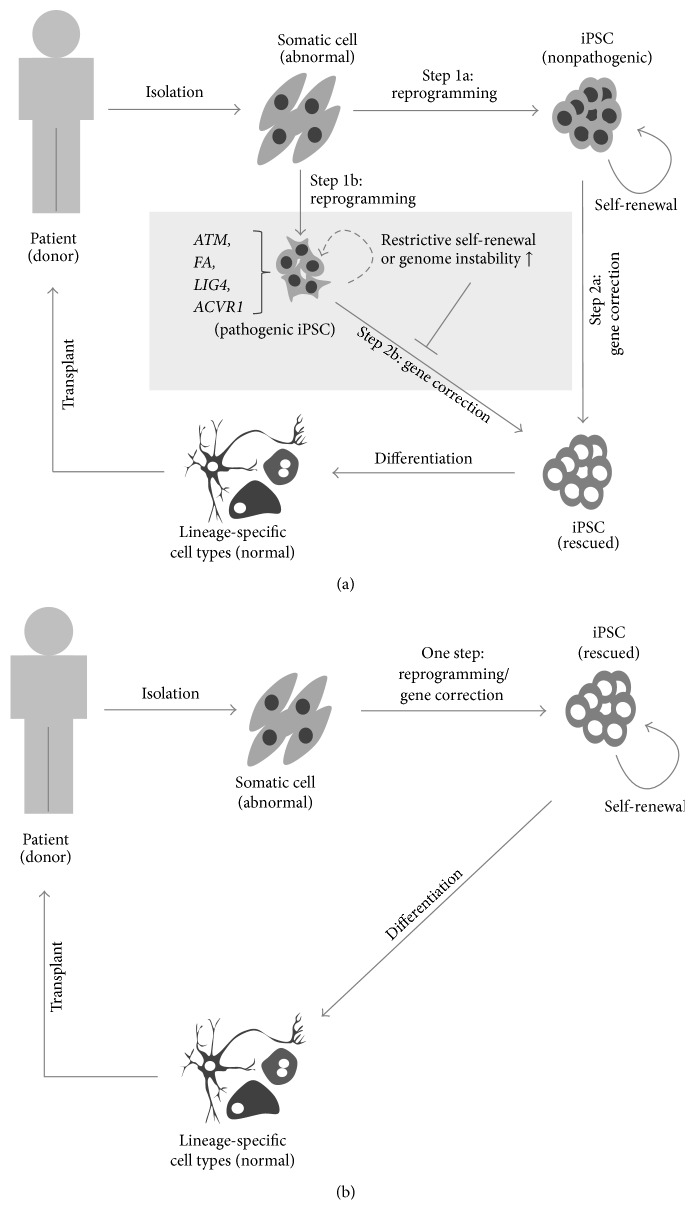
Overview of the combined reprogramming and gene-correction strategy. (a) Current iPSC-based gene-correction approach. Step 1a: nonpathogenic iPSCs can stably maintain their self-renewal property. Step 1b: pathogenic iPSCs, derived from cells with mutations in* ATM*,* FA*,* LIG4*, or* ACVR1*, exhibit restrictive self-renewal and a potential rise of genome instability, hampering progress to Step 2b. Step 2a: the iPSCs generated from Step 1a can be subsequently subject to gene correction with various tools such as ZFNs, TALENs, CRISPR/Cas9, or BAC vectors. (b) A one-step process involving simultaneous reprogramming and gene correction. Gene-corrected iPSCs are directly produced from patient-derived somatic cells, concurrently combining the reprogramming factors and gene-correction tools.

The currently used methods for iPSC-based gene therapy generally involve the application of gene-correction tools in iPSCs after the reprogramming of donor-derived somatic cells (Figure 2(a); Step 1a to Step 2a). However, such methods rely on the assumption that the abnormal gene, which is involved in the disease, has no influence on the generation or maintenance of iPSCs (Figure 2(a); Step 1a). That is, if it were not possible to efficiently generate or maintain iPSCs derived from cells of a patient with one of the aforementioned diseases (Figure 2(a), Step 1b), the current gene therapy method would not be efficiently applicable to the pathogenic iPSCs. Therefore, to overcome this blind spot of conventional gene therapy, the abnormal genes would be first replaced with the normal gene in patient-derived somatic cells, prior to the iPSC stage. This can be accomplished by simultaneously introducing gene-correction tools along with reprogramming factors in the patient-derived somatic cells (Figure 2(b)) [17]. We applied this approach to mALK2 dermal fibroblasts, which exhibit atypical reprogramming and inhibit the maintenance of iPSCs [16, 17]. The expression of reprogramming factors coupled with gene-correction tools, including reprogramming episomal vectors, CRISPR/Cas9-encoding vectors targeting the* ACVR1* gene, and donor DNA carrying the normal base, enabled the generation of normal ALK2-iPSCs without concerns regarding atypical reprogramming or maintenance inhibition caused by the gene mutation [17]. In addition to the application of this approach for vulnerable iPSCs, the one-step generation of gene-corrected iPSCs by simultaneous reprogramming and gene editing has additional advantages of saving time, effort, and cost [17, 51], thereby eliminating the need of performing the cumbersome reprogramming process and then subsequently performing gene editing separately, as required by the conventional gene-correction approach. Therefore, the one-step method described herein is highly practical and extensively applicable to cell-replacement therapies.

### 4.2. Coupled TD and Gene Correction in LRRK2 (G2019S) Somatic Cells

**Figure 3:**
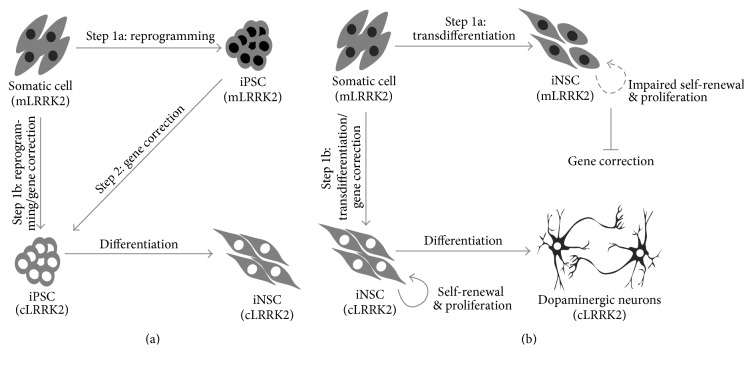
Application of the gene-correction approach in cells with the LRRK2 (G2019S) mutation. (a) Conventional gene-correction approach using mutant iPSCs generated from LRRK2 (G2019S) mutant fibroblasts, involving reprogramming with subsequent gene correction (Step 2 via Step 1a), and the combined reprogramming and gene-correction approach based on LRRK2 (G2019S) mutant fibroblasts (Step 1b). (b) Mutant iNSCs are generated from LRRK2 (G2019S) mutant fibroblasts, potentially exhibiting impaired self-renewal or proliferation in Step 1a, whereas, in Step 1b, transdifferentiation and gene correction occur simultaneously and the corrected iNSCs are capable of stable proliferation with maintained self-renewal potential.

Familial PD caused by genetic mutation is generally rare, but one of the most frequent causes of early- or late-onset PD results from an autosomal dominant mutant of LRRK2 (G2019S), which accounts for 5-6% of all cases of familial PD or 1-2% of sporadic PD cases [52, 53]. And several groups have already demonstrated the ability to genetically correct LRRK2 (G2019S) mutant iPSCs using ZFN-mediated homology directed repair (HDR) [54, 55]. The ZFN-mediated HDR approach could be also performed using LRRK2 (G2019S) somatic cells by combining the reprogramming and gene-correction processes (Figure 3(a), Step 1b). Although this mutation has no effect on the reprogramming process or iPSC maintenance, it can nevertheless help to generate therapeutic iPSCs more rapidly than possible with a two-step generation method, involving reprogramming and subsequent gene correction (Figure 3(a), Step 1a to Step 2). Another attractive property of this approach is that these gene-editing tools can also be applied to iNSCs carrying LRRK2 (G2019S), because one of the main advantages of TD is the lack of risk of teratoma formation, which is a relevant concern with the use of iPSC-derived cells [56], and can rapidly generate lineage-specific cell types. However, the LRRK2 (G2019S) mutation can also cause the progressive degeneration of iNSCs, in which the iNSCs derived from mLRRK2-iPSCs exhibit depletion after undergoing several passages [18]. These findings strongly suggest that mLRRK2 exerts a negative influence on the maintenance of iNSCs derived from mLRRK2-somatic cells. Therefore, to directly generate iNSCs from PD fibroblasts with the LRRK2 (G2019S) mutation which are stably maintained, it is ideal to genetically treat the mLRRK2 fibroblasts during the process of TD, which may be achieved using a coupled TD and gene-correction approach (Figure 3(b), Step 1b). In contrast to the mLRRK2-iNSCs, the gene-corrected iNSCs would be stably self-renewing and differentiate toward dopaminergic neurons (DNs). The main critical factors of gene-corrected stem cells for application in cell-replacement therapy are the ability of continual growth, maintenance of their differentiation potential, and self-renewal ability. Several groups recently introduced methods for the direct conversion of human fibroblasts to DNs [57, 58]. Thus, it could be conceptually possible to generate gene-corrected DNs that are induced directly from PD fibroblasts by coupling gene correction and direct conversion into DNs. However, the induced DNs have a general limitation of continuous growth, which hampers the ability to screen for positive clones as well as to acquire a sufficient amount of therapeutic material. Thus, along with the concurrent reprogramming and gene-correction approach, the application of combined gene correction and TD-mediated iNSC appears to be a suitable approach to generate the therapeutic materials for PD cell therapy.

## 5. Conclusions

To date, numerous types of patient-derived iPSCs have been generated, most of which have been provided as resources for gene therapy. However, there are several diseases for which treatment using current iPSCs-based gene therapy approaches remains a challenge.

Several of these diseases result from chromosomal instability or impaired DNA repair. Although even the generation of low numbers of disease-derived iPSCs is still promising, cases with a high incidence of aneuploidy can be further exacerbated by the reprogramming factors such as OCT4, SOX2, KLF4, and c-MYC. Indeed, ATM-deficient iPSCs acquired serious chromosomal abnormalities after several passages [29]. Although c-MYC, a representative oncoprotein, enhances reprogramming efficiency, it may also substantially increase the probability of abnormalities in reprogrammed cells with a defective DNA repair gene. However, as the proteins involved in DNA repair are normally rescued with the use of the one-step system proposed herein, the reprogramming efficiency could be enhanced, thereby reducing the incidence of aneuploidy.

As the one-step system has been recently introduced, only four cases applying this approach have been reported to date [17, 51, 59]. In all cases, the cells were genetically treated with CRISPR/Cas9-mediated HDR, resulting in a targeting efficiency of 5–17% [17, 51, 59]. This targeting efficiency is comparable to that obtained with iPSC-based approaches, which has been attempted using the diverse gene therapy tools available, such as plasmid, BAC, adeno-associated virus, helper-dependent adenovirus, and ZFN [60].

LRRK2 (G2019S) interacts with lamin B1 protein, which is anchored to the inner nuclear membrane and is involved in breaking the framework of the nuclear envelope, leading to the loss of function of lamin B1 protein, which ultimately increases chromatin instability and eventually depletes the iNSC pool. Therefore, the use of TD-mediated iNSCs coupled with gene correction may enable the maintenance of the chromatin stability of iNSCs, thereby providing a stable therapeutic method for cell replacement in patients with PD.

In addition to gene therapy for the rare diseases mentioned above, the advanced one-step method is generally applicable to all disease-derived cells regardless of the defective gene involved, which can substantially save time, reduce the need for labor-intensive experiments, and cut the cost required for current iPSC-based gene therapy methods by half. Therefore, the one-step method described herein is expected to become increasingly popular for the development of more rapid and personalized cell-replacement therapies in the near future.

## Figures and Tables

**Table 1:** Diseases with the inefficient reprogramming or inhibitory pluripotency maintenance.

Category	Disease	Gene	Gene status	Reprogramming efficiency or pluripotency maintenance	Refs.
DNA repair	Ataxia telangiectasia (A-T)	ATM	7004delCA, 7886delTATTA	Low efficient generation (15% in heterozygote)/extremely low efficient generation (4% in homozygote)	[2]
	Fanconi anemia (FA)	FANCA, FANCD2	del^a^	No reprogramming, or extremely low efficient generation (FANCA), low efficient reprogramming and poor maintenance (FANCD2)	[13, 14]
	FA or breast cancer	BRCA1, BRCA2	ins^a^ in exon 11 or S1598F point mutation for BRCA1, del^a^ in exon 27 for BRCA2	~20-fold lower than normal	[20]
	Cancer	Rad51	Knockdown expression by shRNA	~60-fold lower than normal	[20]
	LIG4 syndrome	LIG4	c.2440C>T in allele 1 and c.1406G>A in allele 2, or c.833G>A	Low reprogramming efficiency (0.002~0.012%) and apoptosis sensitivity	[15]

Signaling	Fibrodysplasia ossificans progressiva (FOP)	ACVR1	c.617G>A	Low reprogramming efficiency (~0.05%) and inhibitory maintenance of iPSCs	[16, 17]

^a^Unknown locus.
